# The PROgnostic ModEl for chronic lung disease (PRO-MEL): development and temporal validation

**DOI:** 10.1186/s12890-024-03233-0

**Published:** 2024-08-30

**Authors:** Sheryl Hui-Xian Ng, Zi Yan Chiam, Gin Tsen Chai, Palvinder Kaur, Wan Fen Yip, Zhi Jun Low, Jermain Chu, Lee Hung Tey, Han Yee Neo, Woan Shin Tan, Allyn Hum

**Affiliations:** 1 Health Services and Outcomes Research, National Healthcare Group, Annex @ National Skin Centre, 1 Mandalay Road, Singapore, 308205 Singapore; 2 Department of Palliative Medicine, Tan Tock Seng Hospital, 11 Jalan Tan Tock Seng, Singapore, 308433 Singapore; 3 Department of Respiratory and Critical Care Medicine, Tan Tock Seng Hospital, 11 Jalan Tan Tock Seng, Singapore, 308433 Singapore; 4 Lee Kong Chian School of Medicine, Nanyang Technological University, 11 Mandalay Road, Singapore, 308232 Singapore; 5 The Palliative Care Centre for Excellence in Research and Education, Dover Park Hospice, 10 Jalan Tan Tock Seng, Singapore, 308436 Singapore

**Keywords:** Lung, Respiratory, Prognostic, Bronchiectasis, Interstitial lung disease, End-of-life

## Abstract

**Background:**

Patients with chronic lung diseases (CLDs), defined as progressive and life-limiting respiratory conditions, experience a heavy symptom burden as the conditions become more advanced, but palliative referral rates are low and late. Prognostic tools can help clinicians identify CLD patients at high risk of deterioration for needs assessments and referral to palliative care. As current prognostic tools may not generalize well across all CLD conditions, we aim to develop and validate a general model to predict one-year mortality in patients presenting with any CLD.

**Methods:**

A retrospective cohort study of patients with a CLD diagnosis at a public hospital from July 2016 to October 2017 was conducted. The outcome of interest was all-cause mortality within one-year of diagnosis. Potential prognostic factors were identified from reviews of prognostic studies in CLD, and data was extracted from electronic medical records. Missing data was imputed using multiple imputation by chained equations. Logistic regression models were developed using variable selection methods and validated in patients seen from January 2018 to December 2019. Discriminative ability, calibration and clinical usefulness of the model was assessed. Model coefficients and performance were pooled across all imputed datasets and reported.

**Results:**

Of the 1000 patients, 122 (12.2%) died within one year. Patients had chronic obstructive pulmonary disease or emphysema (55%), bronchiectasis (38%), interstitial lung diseases (12%), or multiple diagnoses (6%). The model selected through forward stepwise variable selection had the highest AUC (0.77 (0.72–0.82)) and consisted of ten prognostic factors. The model AUC for the validation cohort was 0.75 (0.70, 0.81), and the calibration intercept and slope were − 0.14 (-0.54, 0.26) and 0.74 (0.53, 0.95) respectively. Classifying patients with a predicted risk of death exceeding 0.30 as high risk, the model would correctly identify 3 out 10 decedents and 9 of 10 survivors.

**Conclusions:**

We developed and validated a prognostic model for one-year mortality in patients with CLD using routinely available administrative data. The model will support clinicians in identifying patients across various CLD etiologies who are at risk of deterioration for a basic palliative care assessment to identify unmet needs and trigger an early referral to palliative medicine.

**Trial registration:**

Not applicable (retrospective study).

**Supplementary Information:**

The online version contains supplementary material available at 10.1186/s12890-024-03233-0.

## Introduction

Chronic lung disease (CLD), defined as a group of progressive and life-limiting respiratory conditions, was the third leading cause of death globally in 2017, accounting for 7% of all deaths [1]. Despite the differences in pathophysiology, clinical presentation and prognosis between specific respiratory conditions, majority of CLD patients near the end-of-life experience similar symptoms of dyspnea, cough, fatigue, and psychosocial distress, with a symptom burden comparable to lung cancer [2]. However, only 17–37% of patients with chronic obstructive pulmonary disease (COPD), and 14% of patients with interstitial lung diseases (ILDs) received palliative care for their symptoms and needs before death [3–6].

Difficulty in prognosticating a patient’s disease trajectory stands as one of many barriers to providing timely palliative care in CLD [7]. Despite the recent push to move away from a prognosis-centric referral decision process, [8, 9] prognostic tools can serve as surrogate markers to help non-palliative clinicians identify patients at high risk of short-term deterioration, prioritising them for holistic needs assessments and subsequent referral for integrated support [9, 10]. Moreover, embedding such tools within electronic health records to provide automated flagging of high-risk patients enables prognostication to be easily integrated into existing clinical workflows with minimal incremental workload to the clinicians [11].

However, the heterogeneity in patient characteristics and prognoses within CLD makes prognostication and timely referral to palliative care challenging. This is exacerbated by the existing prognostic tools developed for individual conditions, which cannot be generalised across CLD subtypes. Many prognostic tools have been developed and validated for COPD patients for a variety of outcomes [12–16]. The Gender-Age-Physiology (GAP) model for patients with ILDs, [17, 18] as well as the FACED score and Bronchiectasis Severity Index for patients with bronchiectasis have also been validated for mortality prediction in their respective populations [19]. However, to date, there has not been any model developed for, or validated on CLDs as a group. Furthermore, these tools may incorporate information which may not be collected across all care settings or conditions, such as clinician assessments, lung function measurements or diagnostic investigations [13, 14, 20]. As data on these predictors may not be readily available for all patients, the applicability of these tools in routine clinical practice may be limited.

Development of a prognostic tool generalizable to all patients with CLD would potentially allow for expanded use within a hospital and outpatient clinic setting, with clinicians being able to apply the tool to any patient presenting with CLD of any etiology. To ensure the transferability of the model across care settings, the model would have to be constructed based on routinely available information, such as those recorded in administrative databases. Hence, we aimed to develop and validate a single prognostic model for one-year mortality in patients across various CLDs, using routinely collected data from electronic health records. Our model would then support non-palliative clinicians to identify patients at high risk of deterioration in the next one year to prioritize for holistic needs assessment and subsequent referral for supportive care management.

## Methods

### Study setting

The public healthcare system in Singapore is organized into three geographic healthcare clusters – the National University Health System, the Singapore Health Services, as well as the National Healthcare Group (NHG). Each cluster serves as the regional health manager for approximately 1.5 million residents, [21] and consists of one or more public hospitals providing acute inpatient, specialist outpatient and emergency care, as well as facilities for primary, subacute and long-term care [22]. The public system accounts for almost 80% of all inpatient admissions in Singapore [23]. Tan Tock Seng Hospital (TTSH), where the study population was recruited from, serves a population of a million residents, and is one of the largest public hospitals in the country with more than 1,700 beds [24].

### Study design, study population, and source of data

A retrospective cohort study design was adopted to predict one-year mortality among patients with CLD. Patients were recruited into the study if they had a recorded CLD diagnosis between 1st July 2016 and 31st October 2017 in either the inpatient or specialist outpatient settings within TTSH (Additional File 1). The list of ICD-10CM diagnosis codes indicative of CLD consisted of irreversible progressive respiratory conditions associated with substantial symptom burden towards the end of life and were reviewed by respiratory and palliative clinicians for relevance. COPD patients with a ratio of forced expiratory volume to forced vital capacity exceeding 0.70 in the six months prior were excluded from the study as these patients would not have exhibited persistent airflow obstruction [25].

We extracted patients’ clinical information from electronic health records in TTSH. Death dates, comorbidity information and history of healthcare utilization within NHG were taken from the Regional Health System database, a research database comprising of administrative and billing information (Additional File 2) [26].

### Sample size

Based on a sample size calculation assuming 20 predictors per model with a minimum of 10 events per variable over a one-year period, and a hospital mortality rate of 20%, we recruited 1000 consecutive patients who satisfied the inclusion criteria [27].

### Outcome definition

The date of CLD diagnosis within the study period for each patient was denoted as their index visit date. All-cause mortality within 365 days of the index visit date was the outcome of interest.

### Exposure definition

Potential prognostic factors were shortlisted for inclusion in the model based on a prior scoping review of prognostic factors in non-COPD chronic lung diseases, [28] as well as inputs from respiratory and palliative clinicians. We extracted information on the socio-demographics and prior CLD history as of the index visit. For historical data, we extracted information on low oxygen saturation, need for oxygen therapy or ventilation, as well as biomarkers, echocardiogram findings, functional/physiological and pulmonary assessments six months prior and up to the index visit. Information on comorbidities and previous healthcare utilization was captured up to one year prior.

### Missing data

Only variables with less than 50% missing data were considered for model building. Additionally, to facilitate model convergence, variables with less than 10 decedents’ data were excluded from model building. Data were assumed to be missing at random and multiple imputation using chained equations were used to impute missing data, using information on the revised list of predictors as input for the imputation model (Additional File 2). Categorical variables with two levels were imputed using logistic regression, while variables with more levels were imputed using a multinomial model. Continuous variables were imputed using predictive mean matching. Fifty imputed datasets were then generated.

### Model building

For each imputed dataset, logistic regression models using forward stepwise selection (FW), backward stepwise selection (BW) and least absolute shrinkage and selection operator (LASSO) were built using the list of predictors in **Additional File**2. Under FW, we started with an intercept model with no variables, and added individual variables sequentially until we obtained a final model of the lowest Akaike Information Criterion (AIC) value. Under BW, the initial model contained the entire list of predictors, and individual variables were removed until the model with the lowest AIC value was obtained. With LASSO, the model containing the entire list of predictors was optimized to fit the data, omitting certain predictors in the process [29]. The variables selected using each method were tabulated, and variables that were selected across all 50 datasets were used to construct the models for each modelling approach. We included variables that were included in all 50 datasets, above the recommended 50% threshold [30]. This ensured the final set of selected variables were consistently associated with mortality risk across the imputed datasets, resulting in a parsimonious model for implementation. Model coefficient estimates for each of the modelling approaches were averaged using Rubin’s rules across the 50 imputed datasets.

### Internal validation of model

For each model in each dataset, we assessed discrimination, calibration, and clinical usefulness of the model. Discrimination, the ability of the model to differentiate between decedents from survivors, [31] was assessed using the Area under Curve (AUC), with values closer to one indicating stronger discriminatory ability. Calibration, the extent of agreement between observed outcomes and model predictions, was assessed through the calibration slope and intercept. The calibration intercept quantifies the bias in mean predictions from mean observed data, with values closer to 0 indicating lower bias. The slope, in contrast, provides an assessment of the trend in bias, with values below one suggesting over-estimation by the model, and values above one suggesting under-estimation [32]. Clinical usefulness, the ability of the model to impact decision-making, was assessed through the sensitivity, specificity, as well as positive and negative predictive values at specific risk thresholds, exceeding which patients were defined to be at high risk of death in the next year [31]. A range of thresholds were used to assess the impact of different thresholds on sensitivity of the model [33].

For each imputed dataset, estimates of these performance measures were optimism-corrected over 100 bootstrap iterations. We then applied Rubin’s rule to pool the performance estimates across the 50 imputed datasets [34]. The final model was selected as the model with the best discriminative performance and acceptable model calibration. Additionally, we included stability plots for selected datasets to reflect the stability of our model predictions and performance over 1000 bootstrap iterations [33].

### External validation of model

A second cohort of patients were identified for the external validation of the model. Patients from the validation cohort had at least one inpatient or outpatient visit to TTSH within the period of January 2018 to December 2019, and the same inclusion and exclusion criteria based on diagnosis was applied. Missing data was similarly imputed for 50 datasets. Coefficient estimates of the developed model were applied to these datasets, and model performance estimates and calibration were pooled and reported.

## Results

A total of 1,000 patients with a CLD diagnosis from July 2016 to October 2017 were included in the study. One hundred and twenty-two (12.2%) patients died within one year of the index visit date, with a median (interquartile range) time to death of 132 (47–224) days for decedents. The cohort comprised mostly of patients who had a diagnosis of COPD/emphysema (55%), bronchiectasis (38%), or ILDs (12%), including those with overlapping CLD diagnoses (6%). Most patients were included based on their inpatient diagnosis (40.2%). Only 14 (1.4%) patients had an inpatient palliative referral at the index visit, and 20 (2%) were referred to support services in the community, such as sub-acute and home-based care.

Socio-demographic information, clinical profile and history of healthcare utilization of the cohort can be found in Tables 1 and 2. When compared to survivors in univariate analyses, decedents were more likely to be older (decedents: median = 77.5 years, survivors: 71.5 years), seen as inpatients in the previous six months (32.0% vs. 22.2%), but were less likely to be treated in the specialist outpatient setting (65.6% vs. 76.5%). They were also more likely to have a diagnosis of ILD (decedents: median = 20.5%, survivors: 10.3%), started on long term oxygen therapy (LTOT) (9.0% vs. 1.7%), or have a higher comorbidity burden (decedents: median Charlson Comorbidity Index score = 6, survivors: 4). Only nine (7.4%) decedents had a referral to the inpatient palliative services within one year before death.

**Table 1:** Socio-demographic information and healthcare utilization profile

		All patients (*n* = 1,000)		Survivors (*n* = 878)		Decedents (*n* = 122)		
		*n*	%	*n*	%	*n*	%	*p*-value
**Demographics** **(at index visit)**								
Age (years)	Median (Q1-Q3)	1000	72.0 (64.0–80.0)	878	71.5 (64.0–79.0)	122	77.5 (68.0-83.8)	< 0.001
Sex	Female	333	33.3	290	33.0	43	35.2	0.68
	Male	667	66.7	588	67.0	79	64.8	
Marital status	Married	339	33.9	319	36.3	20	16.4	< 0.001
	Single	34	3.4	31	3.5	3	2.5	
	Divorced/Widowed	19	1.9	18	2.1	1	0.8	
	Unknown	608	60.8	510	58.1	98	80.3	
Ethnic minority*	Minority	189	18.9	160	18.2	29	23.8	0.14
	Majority	811	81.1	718	81.8	93	76.2	
Housing type	1–3 rooms	350	35.0	306	34.9	44	36.1	0.84
	4–5 rooms	406	40.6	356	40.5	50	41.0	
	Executive flats	40	4.0	34	3.9	6	4.9	
	Non-public housing	204	20.4	182	20.7	22	18.0	
Smoking status	Ex-Smoker	92	9.2	83	9.5	9	7.4	0.22
	Non-Smoker	374	37.4	323	36.8	51	41.8	
	Smoker	269	26.9	244	27.8	25	20.5	
	Unknown	265	26.5	228	26.0	37	30.3	
**History of healthcare utilisation** *(up to 6 months prior)*								
1 or more inpatient (emergency) admissions		234	23.4	195	22.2	39	32.0	0.02
1 or more Emergency Department visits		342	34.2	290	33.0	52	42.6	0.04
1 or more Specialist Outpatient Clinic visits		752	75.2	672	76.5	80	65.6	0.01
1 or more polyclinic visits		401	40.1	360	41.0	41	33.6	0.14
1 or more HD/ICU admissions (up to 1 year prior)		61	6.1	55	6.3	6	4.9	0.69

Q1: 25th percentile; Q3: 75th percentile; HD: high dependency; ICU: intensive care unit; *Ethnic majority in Singapore consists of ethnic Chinese, while ethnic minorities include Malays, Indians and other ethnicities

**Table 2:** Clinical profile

		All patients (*n* = 1,000)		Survivors (*n* = 878)		Decedents (*n* = 122)		
		*n*	%	*n*	%	*n*	%	*p*-value
**Lung disease characteristics** **(at index visit)**								
Had a prior CLD diagnosis		622	62.2	539	61.4	83	68.0	0.16
Duration of disease (years)	Median (Q1-Q3)	622	2 (1–5)	539	2 (1–5)	83	2 (1–6)	0.56
Setting of index visit	Inpatient*	402	40.2	325	37.0	77	63.1	< 0.001
	Outpatient^	598	59.8	553	63.0	45	36.9	
*Diagnosis at index visit*								
Bronchitis, emphysema or COPD		550	55.0	498	56.7	52	42.6	< 0.001
Bronchiectasis		383	38.3	328	37.4	55	45.1	0.11
Interstitial pulmonary diseases		115	11.5	90	10.3	25	20.5	< 0.001
> 1 diagnosis at index visit		59	5.9	47	5.4	12	9.8	0.06
*Respiratory complications*								
Chronic respiratory failure		25	2.5	17	1.9	8	6.6	0.01
Pulmonary hypertension or heart disease		13	1.3	8	0.9	5	4.1	0.01
Sequelae of respiratory and unspecified tuberculosis		46	4.6	35	4.0	11	9.0	0.02
**Pulmonary history/parameters** *(up to 6 months prior*,* including index visit)*								
Long term oxygen therapy		26	2.6	15	1.7	11	9.0	< 0.001
Started invasive ventilation		8	0.8	6	0.7	2	1.6	0.25
Started acute non-invasive ventilation		23	2.3	17	1.9	6	4.9	0.05
Long term non-invasive ventilation		3	0.3	3	0.3	0	0.0	1.00
Oxygen saturation < 95%	Yes	115	11.5	86	9.8	29	23.8	< 0.001
	Unknown	188	18.8	171	19.5	17	13.9	
**Biomarkers** *(up to 6 months prior*,* including index visit)*								
Eosinophils, x10^9^/L	Median (Q1-Q3)	656	0.1 (0-0.3)	558	0.1 (0-0.3)	98	0.1 (0-0.3)	0.14
Neutrophils, x10^9^/L	Median (Q1-Q3)	655	6.1 (4.4–8.7)	557	6 (4.3–8.7)	98	6.6 (4.4–8.7)	0.25
MDRD GFR, mL/min/1.73m^2^	Median (Q1-Q3)	690	73.3 (57.1–89.9)	592	73.8 (58.9–90.7)	98	68.9 (42.7–88)	0.03
**Functional/ physiological measurements** *(up to 6 months prior*,* including index visit)*								
Body mass index, Asian categories	18.5 kg/m^2^ or more	575	57.7	536	61.0	39	32.0	0.002
	< 18.5 kg/m^2^	190	19.0	162	18.5	28	22.9	
	Unknown	235	23.5	180	20.5	55	45.1	
1 or more assisted Activity of Daily Living	Yes	131	13.1	90	10.3	41	33.6	< 0.001
	Unknown	464	46.4	424	48.3	40	32.8	
**Comorbidity** *(up to 1 year prior*,* including index visit)*								
Charlson Comorbidity Index score	Median (Q1-Q3)	997	4 (3–7)	875	4 (3–6)	122	6 (4–9)	< 0.001
Renal disease		98	9.8	76	8.7	22	18.0	< 0.001
Diabetes, uncomplicated		166	16.6	139	15.8	27	22.1	0.09
Diabetes, complicated		107	10.7	87	9.9	20	16.4	0.04
Cerebrovascular disease		51	5.1	40	4.6	11	9.0	0.05
Cardiac (CHF, MI, Arrhythmia)		115	11.5	95	10.8	20	16.4	0.09
Cancer		55	5.5	39	4.4	16	13.1	< 0.001
Anaemia		110	11.0	89	10.1	21	17.2	0.03
Hypertension		393	39.3	339	38.6	54	44.3	0.24
Dyslipidaemia		319	31.9	284	32.3	35	28.7	0.47
**Referral destination**								
Inpatient palliative services		11	1.1	2	0.2	9	7.4	< 0.001
Sub-acute care		13	1.3	7	0.8	6	4.9	< 0.001

CLD: chronic lung disease; MDRD GFR: Modification of Diet in Renal Disease glomerular filtration rate; CHF: Congestive heart failure; MI: myocardial infarction; Q1: 25th percentile; Q3: 75th percentile; *includes both emergency attendances and inpatient admissions; ^includes both specialist outpatient visits and day surgeries

We did not include spirometry, echocardiography, several biomarkers and dyspnea scores in model building due to > 70% missing data. (Additional File 3). Applying the three variable selection methods to the imputed datasets, LASSO selected the largest model, with 12 variables, followed by FW and BW (Additional File 4). The model selected via FW was chosen as the final model, having reported the highest AUC at 0.77 (95% CI: 0.72, 0.82); models selected via BW and LASSO reported slightly lower AUCs of 0.74. Based on the calibration intercept and slope estimates, the final model was prone to overpredicting the probability of death (intercept= -0.13, 95% CI: -0.51, 0.24; slope = 0.91, 95% CI: 0.72, 1.10). Calibration and stability plots for selected datasets can be found in Additional File 5 and 6.

The final model consisted of 10 prognostic factors (Table 3). Patients who were older, of male sex or of a minority ethnic group were of higher mortality risk. Other risk factors included being on LTOT, having a history of cancer or a diagnosis of ILD or tuberculosis infection. Having a body mass index below 18.5 kg/m^2^ or requiring assistance with at least one Activity of Daily Living was also associated with a higher mortality risk. In contrast, having specialist outpatient consultations six months prior to diagnosis was associated with lower mortality risk. The equation to compute the risk of death in one year is provided in Additional File 7.

**Table 3:** Final model estimates

Prognostic factor	Pooled Odds ratio (95% CI)
Intercept	0.00 (0.00-0.02)
Age, per year increase	1.03 (1.01–1.06)
Male gender, vs. female gender	1.77 (1.09–2.88)
Minority ethnic group, vs. majority	2.04 (1.21–3.44)
Index diagnosis: interstitial pulmonary diseases, yes vs. no	3.57 (1.95–6.52)
Complication: sequelae of respiratory and unspecified tuberculosis, yes vs. no	3.15 (1.40–7.11)
Started long term oxygen therapy, yes vs. no	4.76 (1.81–12.5)
Most recent body mass index < 18.5 kg/m^2^, yes vs. no	2.95 (1.72–5.05)
At least 1 assisted Activity of Daily Living, yes vs. no	2.96 (1.64–5.32)
History/diagnosis of cancer, yes vs. no	3.50 (1.72–7.13)
History/diagnosis of cerebrovascular disease, yes vs. no	2.63 (1.19–5.80)
History of specialist outpatient visits in 6 months prior, yes vs. no	0.44 (0.27–0.70)

CI: confidence interval

### Internal validation

Sensitivity and specificity of the model at a range of risk thresholds are reported in Table 4. Classifying patients with a predicted probability of death of 0.30 and above as high-risk, sensitivity and specificity of the final model was 37% and 95% respectively, indicating that the model would identify three out of ten dying patients and nine of ten surviving patients correctly. The negative predictive value of the model was 92%, indicating that nine of ten patients would be accurately identified to be at low risk of death.

**Table 4:** Performance measures of final model at internal validation

	Estimate (95% CI)			
AUC	0.77 (0.72, 0.82)			
Intercept	-0.13 (-0.51, 0.24)			
Slope	0.91 (0.72, 1.10)			
	**Threshold for high risk of mortality**			
	**0.10**	**0.30**	**0.50**	**0.70**
Sensitivity	76%	37%	15%	4%
Specificity	68%	95%	99%	100%
Positive predictive value	25%	48%	66%	59%
Negative predictive value	95%	92%	89%	88%

AUC: Area under curve; CI: confidence interval

### External validation

Of the 500 patients in the external validation (EV) cohort, 97 (19.4%) died within one year of the reference visit date. A comparison of the development and EV cohorts can be found in Additional File 8. In brief, compared to the development cohort, there were more patients admitted via the emergency department or inpatient setting (52.4% vs. 40.2%) in the EV cohort. The EV cohort comprised a larger proportion of patients diagnosed with bronchitis, emphysema, or COPD (80.6% vs. 55.0%), and a smaller proportion of patients with bronchiectasis (19.4% vs. 38.3%) or ILDs (5.6% vs. 11.5%). More patients in the EV cohort had an inpatient admission in the one year prior (59.2% vs. 36.8%), as well as a history of CLD in the past five years (81.8% vs. 64.6%). The Charlson Comorbidity Index score was also higher in the EV cohort compared to the development cohort (median (interquartile range): 5 (4–8) vs. 4 (3–7)).

Applying the coefficients of the final model on the EV cohort, the AUC of the model in the EV cohort was similar at 0.75 (0.70, 0.81), while the extent of overprediction increased slightly with calibration intercept and slope of -0.14 (-0.54, 0.26) and 0.74 (0.53, 0.95) respectively. Sensitivity of the model at the threshold of 0.30 as high risk remained similar at 38%, while the negative predictive value reduced slightly to 86% (Table 5).

**Table 5:** Pooled model performance measures at external validation

	Estimate (95% CI)			
AUC	0.75 (0.70, 0.81)			
Intercept	-0.14 (-0.54, 0.26)			
Slope	0.74 (0.53, 0.95)			
	**Threshold for high risk of mortality**			
	**0.10**	**0.30**	**0.50**	**0.70**
Sensitivity	82%	38%	17%	5%
Specificity	58%	89%	97%	99%
Positive predictive value	32%	45%	55%	58%
Negative predictive value	93%	86%	83%	81%

AUC: Area under curve; CI: confidence interval

## Discussion

### Main findings

We developed and validated the PROgnostic ModEl for Chronic Lung Disease (PRO-MEL), a model consisting of ten prognostic factors to predict one-year mortality in patients with CLDs. The performance of PRO-MEL remained robust in the validation cohort. Patients identified to be at high-risk of death in the next year by PRO-MEL should be prioritized for needs assessments and subsequent palliative referrals.

The prognostic factors in PRO-MEL corroborate with the wider literature on prognostic factors and indices in lung diseases. Our model incorporated four of ten risk factors identified in a recent systematic review of prognostic factors in COPD: older age, male sex, lower body mass index (BMI), and history of LTOT [35, 36]. Older age and male sex are the components of the GAP and ILD-GAP indices for patients with idiopathic pulmonary fibrosis (IPF) and ILDs, [17, 18] with BMI also included in prognostic indices for prediction of mortality in ILD [37] and bronchiectasis patients [38, 39]. The association of an ILD diagnosis with higher risk of death was consistent with observations of poorer outcomes in patients with ILD compared to a similar group with COPD [40, 41]. The association of a prior history of cancer and stroke with higher mortality risks were expected as cancer and stroke are common causes of death in COPD patients [36]. Poorer functional status depicted as requiring assistance with ADLs has also been suggested to be a marker of disease severity with poorer outcomes in COPD [42].

However, there were established prognostic factors that were absent from our model. Overlapping syndromes, or having multiple respiratory conditions, were not associated with higher mortality risk in the final model, despite other studies noting poorer outcomes in patients with co-occurring COPD, bronchiectasis and ILD [43, 44]. Smoking history was not selected as a prognostic factor, despite being associated with poorer outcomes in both COPD and ILD [45]. The lack of association of these two prominent risk factors with mortality risk in our cohort could possibly be attributed to their similarity in frequency amongst survivors and decedents. The same could also be said about having a prior history of diabetes and cardiovascular disease. While having a recent readmission was not predictive of mortality in our study, having prior outpatient visits was associated with lower mortality risk, reflecting the pertinence of prior healthcare utilization as a proxy of disease severity in CLD. Notably, we also did not assess the role of lung function in predicting mortality due to high missingness of data. While lung function is strongly predictive of patient prognosis in CLD, [14, 28] patients with advanced lung diseases may not be able to perform lung function satisfactorily, limiting its utility in our model intended for application on all CLD patients [46].

The performance of PRO-MEL is comparable to published estimates for disease-specific models for CLD. The AUC of PRO-MEL, at 0.77, was on par with estimates reported for validated prognostic indices for COPD and ILD [35, 47]. The calibration intercept and slope suggested slight over-estimation of predicted mortality risks, but previous validation studies on the GAP index have reported similarly poor calibration, [47, 48] and this metric has not been reported for any of the indices for COPD, [35] preventing comparison. While model sensitivity, specificity, PPV and NPV are hardly reported in other studies, PRO-MEL has exhibited consistently high specificity and NPV across most risk thresholds, indicating that the model will be accurate in identifying patients who are at low-risk of mortality. However, as a diagnostic tool, the sensitivity and PPV of the model should be adequately high to ensure most high-risk patients are correctly identified. Lowering the threshold may increase the sensitivity of the model but would also result in a larger volume of patients to be identified, screened and referred for specialist palliative care. Our selected risk threshold at 0.30 balanced both model sensitivity and the impact on resources to ensure that patients in need of palliative care are adequately identified without overburdening the screening and palliative teams. Furthermore, considering the current referral rates of 6% in the validation cohort and 14-37% in studies elsewhere, PRO-MEL, at a risk threshold of 0.30, would nonetheless facilitate the identification, assessment, and subsequent referral of patients at a rate outperforming the status quo [3–6].

Validation of PRO-MEL on a more recent cohort of patients with a different mix of CLDs showed similar discriminative performance, but poorer calibration. The model was able to perform reasonably well despite the external validation cohort having a higher mortality rate as well as more patients with COPD, bronchitis or emphysema. PRO-MEL will nonetheless require recalibration through updating of the intercept or a complete reweighting of the predictors to reduce the bias in predictions before use [32]. As patient population characteristics inevitably vary over time, monitoring of model performance, as well as regular recalibration and updating will be necessary as part of model implementation [49, 50]. A separate prospective validation study will be conducted to formally establish the transportability of the model to other settings or over time, [49] and evaluate the impact of the model on clinical practice [51].

Adopting a pragmatic approach with a general prognostic model for CLD would provide non-palliative physicians with a tool to identify those with higher mortality risk across all different etiologies. Current disease-specific prognostic models suffer from the limitation of only predicting mortality risk for diseases that were included in their respective derivative cohort. This excludes patients with other forms of CLDs, such as those with overlapping pathologies or rare lung diseases. For instance, up to 67% of the patients with IPF may have co-existing emphysema, and to date, there are no prognostic tool that uses routinely available clinical parameters for these patients [52]. Despite the good discriminative performance of the ILD-GAP index in common ILDs such as IPF and connective tissue disease-related ILDs, [53, 54] physicians often struggle to prognosticate other forms of rare ILDs [18]. A general prognostic model will be helpful in such instances to alert the managing physician to perform a needs assessment and consider early referral to palliative care.

Moreover, PRO-MEL will be useful to support non-palliative clinicians in prospectively identifying patients likely to deteriorate in the short term, allowing for timely access to quality patient care to address their needs along their subsequent disease trajectory [11]. Kaur et al. demonstrated that patients with advanced respiratory disease incur substantial healthcare expenditure and utilization in the last year of life, with a sharp increase in the last months of life, mainly driven by inpatient admissions, [55] suggesting that these patients would benefit from early access to palliative care to address their needs. By identifying patients who are at high risk of death within the next year, those likely to deteriorate and experience exacerbations or complications in the coming months could receive both disease-modifying and palliative treatment concurrently [56]. Compared with patients with lung cancer, patients with COPD have more frequent and longer stays in intensive care, and were less likely to receive medication for symptom palliation [57]. Patients with bronchiectasis or ILD were less likely than patients with COPD to receive palliative support [58]. The early identification of high-risk patients for review and assessment would improve timeliness of referrals and access to palliative care for these patients, [9] and mitigate the escalation of healthcare utilization and expenditure nearing the end of life [59]. Introduction of primary palliative care concurrent with disease-specific treatment will focus on improving their quality of life and wellbeing, while supporting them in managing their symptoms, as well as the emotional, social and spiritual aspects of their disease [10].

### Strengths and limitations

Strengths of our study include the adoption of best practices in model development methodology. Prognostic factors included in our study were incorporated from prior systematic reviews on prognostic factors in COPD, and a scoping review on non-COPD prognostic factors the team conducted [13, 14, 28]. Candidate predictors were also screened for availability of data to ensure that the information would be routinely available in most patients and not be exclusive to specific healthcare settings, thereby expanding the generalizability of the model. We also conducted multiple imputations of missing data for 50 datasets, and shortlisted predictors which were applicable to all imputed datasets. This ensured that only predictors that were always associated with mortality across all the datasets were selected, maintaining the parsimony of the final model. In addition to internal validation, we also performed an additional temporal validation to ensure the model performance was reliable in a separate, more recent cohort of patients. Lastly, the model uses routinely available data in administrative billing databases, increasing the transferability of the model to other health systems.

Limitations of our study include the use of data from only one of the three public healthcare clusters in Singapore, which may limit the generalizability of our findings to other health systems throughout the world. However, the model was constructed on an ethnically diverse population, and the role of ethnicity was reflected as the disparity between the ethnic majority and minority groups, increasing its applicability to other health systems with a similar multi-ethnic demographic structure. Furthermore, as most of the predictors in PRO-MEL are related to diagnoses and prior healthcare utilization, we expect the model to maintain reasonable utility in identifying patients at high risk of mortality in other populations. As we used ICD-10CM diagnosis codes to identify patients and did not have access to complete spirometry, dyspnea or exacerbation data, we were unable to stage the disease to identify patients of greater disease severity. Furthermore, we did not incorporate spirometry, history of exacerbations, or other measures of dyspnea in the model, as data was not routinely available for all patients. Lastly, as this was a retrospective database study, the model was developed on historical data, and would require validation in a subsequent prospective study to ensure its utility in clinical practice. Lastly, as we validated the model on a newer cohort of patients from the same health system in Singapore, we were unable to demonstrate if the model will perform similarly well in other health systems. Further external validation studies on independent populations and settings are required.

## Conclusion

We developed and validated a one-year mortality prediction model, PRO-MEL, in patients with CLD using routinely available administrative data. PRO-MEL will support non-palliative clinicians across different settings and specialties in identifying patients earlier at their disease trajectory but at high risk of deterioration for a comprehensive basic palliative care assessment to identify unmet needs. This allows for earlier support of their psycho-emotional and social needs, and thereby improving their quality of life. Future work will assess the clinical usefulness of PRO-MEL as a mortality risk prediction algorithm embedded within electronic medical record systems, as part of a clinical workflow for identification, assessment, and management of patients with non-malignant chronic respiratory disease.

### Electronic supplementary material

Below is the link to the electronic supplementary material.

Supplementary Material 1

Supplementary Material 2

Supplementary Material 3

Supplementary Material 4

Supplementary Material 5

Supplementary Material 6

Supplementary Material 7

Supplementary Material 8

Supplementary Material 9

## Data Availability

The datasets generated and/or analysed during the current study are not publicly available due to NHG’s data protection policies and restrictions imposed by the ethics committee to ensure data privacy of patients. Aggregate data may be made available from the corresponding author on reasonable request.

## Abbreviations

AUC: Area Under Curve
BMI: Body mass index
BW: Backward stepwise
CLD: Chronic lung disease
COPD: Chronic obstructive pulmonary disease
EV: External validation
FW: Forward stepwise
GAP: Gender-Age-Physiology
ILD: Interstitial lung disease
IPF: Idiopathic pulmonary fibrosis
LASSO: Least absolute shrinkage and selection operator
LTOT: Long term oxygen therapy
NHG: National Healthcare Group
PRO-MEL: PROgnostic ModEl for Chronic Lung Disease
TTSH: Tan Tock Seng Hospital
