# Supplementary material for: The PROgnostic ModEl for chronic lung disease (PRO-MEL): development and temporal validation
Source: BMC Pulm Med. 2024 Aug 30;24:429. doi: 10.1186/s12890-024-03233-0 (PMC11365240; doi:10.1186/s12890-024-03233-0)
Supplement: Supplementary file 1 — Supplementary Material 1 [file 12890_2024_3233_MOESM1_ESM.docx]

# Additional File 1. List of ICD-10CM diagnosis codes classified as chronic lung disease

| S/N | ICD Codes | Disease |
| --- | --- | --- |
| 1 | J41 | Simple and mucopurulent chronic bronchitis |
|  | J41.0 | Simple chronic bronchitis |
|  | J41.1. | Mucopurulent chronic bronchitis |
|  | J41.8 | Mixed simple and mucopurulent chronic bronchitis |
| 2 | J42 | Unspecified chronic bronchitis |
| 3 | J43 | Emphysema |
|  | J43.1 | Panlobular emphysema |
|  | J43.2 | Centrilobular emphysema |
|  | J43.8 | Other emphysema |
|  | J43.9 | Emphysema, unspecified |
| 4 | J44 | Other chronic obstructive pulmonary disease |
|  | J44.0 | Chronic obstructive pulmonary disease with acute lower respiratory infection |
|  | J44.1 | Chronic obstructive pulmonary disease with (acute) exacerbation |
|  | J44.9 | Chronic obstructive pulmonary disease, unspecified |
| 5 | J47 | Bronchiectasis |
|  | J47.0 | Bronchiectasis with acute lower respiratory infection |
|  | J47.1 | Bronchiectasis with (acute) exacerbation |
|  | J47.9 | Bronchiectasis, uncomplicated |
| 6 | J60 | Coalworker's pneumoconiosis |
|  | J61 | Pneumoconiosis due to asbestos and other mineral fibers |
|  | J62 | Pneumoconiosis due to dust containing silica |
|  | J63 | Pneumoconiosis due to other inorganic dusts |
|  | J64 | Unspecified pneumoconiosis |
|  | J65 | Pneumoconiosis associated with tuberculosis |
|  | J67 | Hypersensitivity pneumonitis due to organic dust |
|  | J67.0 | Farmer's lung |
|  | J67.2 | Bird fancier's lung |
|  | J67.8 | Hypersensitivity pneumonitis due to other organic dusts |
|  | J67.9 | Hypersensitivity pneumonitis due to unspecified organic dust |
| 7 | J68.4 | Chronic respiratory conditions due to chemicals, gases, fumes and vapors |
| 8 | J70.3 | Chronic drug-induced interstitial lung disorders |
|  | J70.4 | Drug-induced interstitial lung disorders, unspecified |
| 9 | J84 | Other interstitial pulmonary diseases |
| 10 | J84.1 | Other interstitial pulmonary diseases with fibrosis |
|  | J84.10 | Pulmonary fibrosis, unspecified |
|  | J84.11 | Idiopathic interstitial pneumonia |
|  | J84.111 | Idiopathic interstitial pneumonia, not otherwise specified |
|  | J84.112 | Idiopathic pulmonary fibrosis |
|  | J84.113 | Idiopathic non-specific interstitial pneumonitis |
|  | J84.114 | Acute interstitial pneumonitis |
|  | J84.115 | Respiratory bronchiolitis interstitial lung disease |
|  | J84.116 | Cryptogenic organizing pneumonia |
|  | J84.117 | Desquamative interstitial pneumonia |
|  | J84.17 | Other interstitial pulmonary diseases with fibrosis in diseases classified elsewhere |
|  | J84.8 | Other specified interstitial pulmonary diseases |
|  | J84.81 | Lymphangioleiomyomatosis |
|  | J84.82 | Adult pulmonary Langerhans cell histiocytosis |
|  | J84.89 | Other specified interstitial pulmonary diseases |
|  | J84.9 | Interstitial pulmonary disease, unspecified |
| 11 | J96 | Respiratory failure, not elsewhere classified |
|  | J96.1 | Chronic respiratory failure |
|  | J96.10 | Chronic respiratory failure, unspecified whether with hypoxia or hypercapnia |
|  | J96.11 | Chronic respiratory failure with hypoxia |
|  | J96.12 | Chronic respiratory failure with hypercapnia |
|  | J96.2 | Acute and chronic respiratory failure |
|  | J96.20 | Acute and chronic respiratory failure, unspecified whether with hypoxia or hypercapnia |
|  | J96.21 | Acute and chronic respiratory failure with hypoxia |
|  | J96.22 | Acute and chronic respiratory failure with hypercapnia |
|  | J96.9 | Respiratory failure, unspecified |
|  | J96.90 | Respiratory failure, unspecified, unspecified whether with hypoxia or hypercapnia |
|  | J96.91 | Respiratory failure, unspecified with hypoxia |
|  | J96.92 | Respiratory failure, unspecified with hypercapnia |
| 12 | J98.2 | Interstitial emphysema |
| 13 | D86 | Sarcoidosis |
|  | D86.0 | Sarcoidosis of lung |
|  | D86.2 | Sarcoidosis of lung with sarcoidosis of lymph nodes |
| 14 | M05.1 | Rheumatoid lung disease with rheumatoid arthritis, also includes M05.10 to M05.19 |
| 15 | M32.13 | Lung involvement in systemic lupus erythematosus |
| 16 | M33.11 | Other dermatomyositis with respiratory involvement |
|  | M33.21 | Polymyositis with respiratory involvement |
|  | M33.91 | Dermatopolymyositis, unspecified with respiratory involvement |
| 17 | M34.81 | Systemic sclerosis with lung involvement |
| 18 | M35.02 | Sicca syndrome with lung involvement |
| 19 | I27.2 | Other secondary pulmonary hypertension |
|  | I27.20 | Pulmonary hypertension, unspecified |
|  | I27.23 | Pulmonary hypertension due to lung diseases and hypoxia |
| 20 | I27.81 | Cor pulmonale (chronic) |
|  | I27.9 | Pulmonary heart disease, unspecified |
| 21 | B90.9 | Sequelae of respiratory and unspecified tuberculosis |
