# Supplementary material for: The PROgnostic ModEl for chronic lung disease (PRO-MEL): development and temporal validation
Source: BMC Pulm Med. 2024 Aug 30;24:429. doi: 10.1186/s12890-024-03233-0 (PMC11365240; doi:10.1186/s12890-024-03233-0)
Supplement: Supplementary file 2 — Supplementary Material 2 [file 12890_2024_3233_MOESM2_ESM.docx]

# Additional File 2. List of potential prognostic factors extracted from databases

| **Variable** | **Variable type** | **Specification** | **Included for model building** | **Reason for exclusion** |
| --- | --- | --- | --- | --- |
| **Demographics** (at index visit) | | | | |
| Age | Continuous | (Years) | √ |  |
| Gender | Categorical | Male / Female | √ |  |
| Race | Categorical | Minority / Majority | √ |  |
| Housing type | Categorical | 1-3 rooms / 4-5 rooms / Executive / Private and others | √ |  |
| Smoking status | Categorical | Ex-Smoker / Non-Smoker / Smoker | √* |  |
| Marital status | Categorical | Single / Married/ Divorced/Widowed / Unknown |  | High missingness of data |
|  |  |  |  |  |
| **Lung disease characteristics** (at index visit) | | | | |
| Setting of index visit | Categorical | Inpatient / Outpatient |  | Design of model to be setting-agnostic |
| Length of stay of index visit | Continuous | (Days) |  | Sparse data |
| Prior CLD diagnosis | Categorical | Yes/No | √ |  |
| Duration of disease | Continuous | (Years) | √ |  |
| More than 1 CLD diagnosed | Categorical | Yes/No | √ |  |
|  |  |  |  |  |
| *Index diagnosis: Subtype of CLD* |  |  |  |  |
| Chronic obstructive pulmonary disease/emphysema | Categorical | Yes/No | √ |  |
| Bronchiectasis | Categorical | Yes/No | √ |  |
| Interstitial pulmonary diseases | Categorical | Yes/No | √ |  |
|  |  |  |  |  |
| *Index diagnosis: Complication of CLD* |  |  |  |  |
| Chronic respiratory failure | Categorical | Yes/No | √ |  |
| Sequelae of respiratory and unspecified tuberculosis | Categorical | Yes/No | √ |  |
| Pulmonary hypertension or heart disease | Categorical | Yes/No | √ |  |
|  |  |  |  |  |
| **Pulmonary history/parameters** (Up to 6 months prior, including index visit) | | | | |
| MMRC Dyspnea Score ≥2 | Categorical | Yes/No |  | High missingness of data |
| Oxygen saturation <95% | Categorical | Yes/No | √* |  |
| Started long term oxygen therapy | Categorical | Yes/No | √ |  |
| Started invasive ventilation | Categorical | Yes/No |  | Sparse data |
| Started acute invasive ventilation | Categorical | Yes/No |  | Sparse data |
| Started long term non-invasive ventilation | Categorical | Yes/No |  | Sparse data |
| Exacerbation associated with COPD or bronchiectasis within 3 months prior | Categorical | Yes/No |  | Sparse data |
| Pneumonia associated with a respiratory disease within 3 months prior | Categorical | Yes/No |  | Sparse data |
| Predicted forced expiratory volume in one second (pre) | Continuous | (%) |  | High missingness of data |
| Predicted forced expiratory volume in one second (post) | Continuous | (%) |  | High missingness of data |
| Predicted forced vital capacity (pre) | Continuous | (%) |  | High missingness of data |
| Predicted forced vital capacity (post) | Continuous | (%) |  | High missingness of data |
| Predicted diffusion capacity of lung for carbon monoxide | Continuous | (%) |  | High missingness of data |
|  |  |  |  |  |
| **Biomarkers** (Up to 6 months prior, including index visit) | | | | |
| Eosinophils | Continuous | (x10^9^/L) | √* |  |
| Neutrophils | Continuous | (x10^9^/L) | √* |  |
| MDRD GFR | Continuous | (mL/min/1.73m^2^) | √* |  |
| Albumin | Continuous | (g/L) |  | High missingness of data |
| C-reactive protein | Continuous | (mg/L) |  | High missingness of data |
| Natriuretic peptide | Continuous | (pg/mL) |  | High missingness of data |
| Carcinoembryonic antigen | Continuous | (µg/L) |  | High missingness of data |
| Ferritin | Continuous | (µg/L) |  | High missingness of data |
| High-density lipoprotein | Continuous | (mmol/L) |  | High missingness of data |
| Low-density lipoprotein | Continuous | (mmol/L) |  | High missingness of data |
| Triglyceride | Continuous | (mmol/L) |  | High missingness of data |
| Creatinine | Continuous | (µmol/L) |  | High missingness of data |
| Pulmonary artery systolic pressure | Continuous | (mmHg) |  | High missingness of data |
|  |  |  |  |  |
| **Functional/ physiological measurements** (Up to 6 months prior, including index visit) | | | | |
| 6-minute walk distance | Continuous | (Metres) |  | High missingness of data |
| Body mass index, Asian categories | Categorical | 10 to <18.5 kg/m^2^ / 18.5 to <23 kg/m^2^ / 23 to <27.5 kg/m^2^ / ≥27.5 kg/m^2^ | √* |  |
| At least 1 assisted Activities of Daily Living | Categorical | Yes/No | √* |  |
|  |  |  |  |  |
| **Utilization** (Up to 6 months prior) |  |  |  |  |
| History of inpatient (emergency) admissions | Categorical | Yes/No | √ |  |
| History of Emergency Department visits | Categorical | Yes/No | √ |  |
| History of Specialist Outpatient Clinic visits | Categorical | Yes/No | √ |  |
| History of polyclinic visits | Categorical | Yes/No | √ |  |
|  |  |  |  |  |
| Ever admitted to high dependency/ intensive care unit (up to 1 year prior) | Categorical | Yes/No | √ |  |
|  |  |  |  |  |
| **Comorbidity** (Up to 1 year prior, including index visit) | | | | |
| Charlson Comorbidity Index score | Continuous | (No units) |  | Collinearity with categorical variables |
| *History/diagnosis of comorbid conditions* |  |  |  |  |
| Renal disease | Categorical | Yes/No | √ |  |
| Diabetes, uncomplicated | Categorical | Yes/No | √ |  |
| Diabetes, complicated | Categorical | Yes/No | √ |  |
| Cerebrovascular disease | Categorical | Yes/No | √ |  |
| Cardiac conditions | Categorical | Yes/No | √ |  |
| Cancer | Categorical | Yes/No | √ |  |
| Anaemia | Categorical | Yes/No | √ |  |
| Dyslipidaemia | Categorical | Yes/No | √ |  |
| Hypertension | Categorical | Yes/No | √ |  |
| Dementia | Categorical | Yes/No |  | Sparse data |
| Connective tissue/rheumatic disease | Categorical | Yes/No |  | Sparse data |
| Peripheral vascular disease | Categorical | Yes/No |  | Sparse data |
| Hemi/paraplegia | Categorical | Yes/No |  | Sparse data |
| Peptic ulcer disease | Categorical | Yes/No |  | Sparse data |
| Liver disease, mild and moderate | Categorical | Yes/No |  | Sparse data |
| Deep vein thrombosis | Categorical | Yes/No |  | Sparse data |
| Obstructive sleep apnea | Categorical | Yes/No |  | Sparse data |
| Pulmonary embolism | Categorical | Yes/No |  | Sparse data |
| Pulmonary hypertension | Categorical | Yes/No |  | Sparse data |

CLD: end-stage lung disease; MDRD GFR: Modification of Diet in Renal Disease glomerular filtration rate; *missing data was multiply imputed before use
