# Supplementary material for: The PROgnostic ModEl for chronic lung disease (PRO-MEL): development and temporal validation
Source: BMC Pulm Med. 2024 Aug 30;24:429. doi: 10.1186/s12890-024-03233-0 (PMC11365240; doi:10.1186/s12890-024-03233-0)
Supplement: Supplementary file 3 — Supplementary Material 3 [file 12890_2024_3233_MOESM3_ESM.docx]

# Additional File 3. Measures of disease severity and biomarkers

|  |  | **All patients (n=1000)** | |  | **Survivors (n=878)** | | **Decedents (n=122)** | |  |
| --- | --- | --- | --- | --- | --- | --- | --- | --- | --- |
|  |  | n | % |  | n | % | n | % | p-value |
| **Dyspnea** |  |  |  |  |  |  |  |  |  |
| MMRC Dyspnea Score ≥2 | Yes | 123 | 12.3 |  | 116 | 13.2 | 7 | 5.7 | 0.08 |
|  | NA | 780 | 78.0 |  | 666 | 75.9 | 114 | 93.4 |  |
|  |  |  |  |  |  |  |  |  |  |
|  |  | **n** | **Median (Q1-Q3)** |  | **n** | **Median (Q1-Q3)** | **n** | **Median (Q1-Q3)** | **p-value** |
| **Spirometry**  *(Index visit + most recent admission in 6 months prior)* |  |  |  |  |  |  |  |  |  |
| Predicted forced expiratory volume in one second (pre), % |  | 266 | 54 (41-71) |  | 247 | 54 (41-70) | 19 | 63 (33.5-88) | 0.42 |
| Predicted forced expiratory volume in one second (post), % |  | 216 | 57 (43-73) |  | 200 | 57 (43.8-73) | 16 | 50 (27.8-69.5) | 0.24 |
| Predicted forced vital capacity (pre), % |  | 266 | 73 (57-89.8) |  | 247 | 73 (58.5-90) | 19 | 52 (43-81.5) | 0.04 |
| Predicted forced vital capacity (post), % |  | 216 | 77.5 (59.8-94) |  | 200 | 78 (62-94) | 16 | 52.5 (34.8-66.8) | <0.001 |
| Predicted diffusion capacity of lung for carbon monoxide, % |  | 73 | 47 (37-56) |  | 66 | 48 (37-55.8) | 7 | 44 (39.5-54) | 0.93 |
|  |  |  |  |  |  |  |  |  |  |
|  |  |  |  |  |  |  |  |  |  |
| **Biomarkers**  *(Index visit + most recent admission in 6 months prior)* |  | **n** | **Median (Q1-Q3)** |  | **n** | **Median (Q1-Q3)** | **n** | **Median (Q1-Q3)** | **p-value** |
| Albumin, g/L |  | 478 | 34.0 (30.0-36.0) |  | 392 | 34.0 (31.0-37.0) | 86 | 30.0 (26.0-34.0) | <0.001 |
| Eosinophils, x10^9^/L |  | 656 | 0.1 (0-0.3) |  | 558 | 0.1 (0-0.3) | 98 | 0.1 (0-0.3) | 0.14 |
| Neutrophils, x10^9^/L |  | 655 | 6.1 (4.4-8.7) |  | 557 | 6.0 (4.3-8.7) | 98 | 6.6 (4.4-8.7) | 0.25 |
| C-reactive protein, mg/L |  | 416 | 20.2 (4.1-58.1) |  | 343 | 17.0 (3.9-58.0) | 73 | 27.0 (7.7-59.2) | 0.29 |
| Natriuretic peptide, pg/mL |  | 104 | 172.0 (46.8-414.5) |  | 83 | 137.0 (44.5-373.0) | 21 | 261.0 (210.0-725.0) | 0.01 |
| Carcinoembryonic antigen, µg/L |  | 10 | 5.5 (3.2-7.0) |  | 9 | 6.0 (4.0-7.0) | 1 | 2.0 (2.0-2.0) | 0.29 |
| Ferritin, µg/L |  | 127 | 193.0 (94.0-330.5) |  | 90 | 192.0 (89.2-305.5) | 37 | 229.0 (98.0-401.0) | 0.56 |
| High-density lipoprotein, mmol/L |  | 165 | 1.2 (0.9-1.5) |  | 150 | 1.2 (1.0-1.5) | 15 | 1.3 (0.9-1.4) | 0.63 |
| Low-density lipoprotein, mmol/L |  | 164 | 2.2 (1.7-2.7) |  | 150 | 2.2 (1.7-2.7) | 14 | 2.1 (1.7-2.8) | 0.83 |
| Triglyceride, mmol/L |  | 164 | 0.9 (0.7-1.3) |  | 150 | 1.0 (0.7-1.3) | 14 | 0.8 (0.7-1.0) | 0.12 |
| Creatinine, µmol/L |  | 691 | 81.0 (66.0-105.0) |  | 593 | 80.0 (66.0-103.0) | 98 | 87.5 (66.2-120.5) | 0.04 |
| MDRD GFR, mL/min/1.73m2 |  | 690 | 73.3 (57.1-89.9) |  | 592 | 73.8 (58.9-90.7) | 98 | 68.9 (42.7-88.0) | 0.03 |
| Pulmonary artery systolic pressure, mmHg |  | 17 | 44.0 (38.8-58.0) |  | 11 | 44 (35.9-57.2) | 6 | 49.2 (39.2-60.2) | 0.69 |

MDRD GFR: Modification of Diet in Renal Disease glomerular filtration rate; mMRC: Modified Medical Research Council; Q1: 25^th^ percentile; Q3: 75^th^ percentile
