# Supplementary material for: The PROgnostic ModEl for chronic lung disease (PRO-MEL): development and temporal validation
Source: BMC Pulm Med. 2024 Aug 30;24:429. doi: 10.1186/s12890-024-03233-0 (PMC11365240; doi:10.1186/s12890-024-03233-0)
Supplement: Supplementary file 4 — Supplementary Material 4 [file 12890_2024_3233_MOESM4_ESM.docx]

# Additional File 4. Models selected through different variable selection methods

|  | **Variable** | **Backward stepwise** | **Forward stepwise** | **LASSO** |
| --- | --- | --- | --- | --- |
|  |  | **OR (95% confidence interval)** | | |
| **Demographics** | Age, per year increase | 1.03 (1.00-1.05) | 1.03 (1.01-1.06) | 1.02 (1.00-1.05) |
|  | Male gender, vs female gender | - | 1.77 (1.09-2.88) | - |
|  | Minority ethnic group, vs majority | 1.91 (1.14-3.19) | 2.04 (1.21-3.44) | 1.88 (1.12-3.17) |
| **Lung disease characteristics** | Index diagnosis: interstitial pulmonary diseases, yes vs no | - | 3.57 (1.95-6.52) | - |
|  | Complication: sequelae of respiratory and unspecified tuberculosis, yes vs no | - | 3.15 (1.40-7.11) | - |
| **Pulmonary history/parameters** | Most recent oxygen saturation <95%, yes vs no | - | - | 1.67 (0.92-3.03) |
|  | Started long term oxygen therapy, yes vs no | 4.34 (1.72-10.93) | 4.76 (1.81-12.5) | 2.93 (1.09-7.92) |
| **Functional/ physiological measurements** | Most recent body mass index<18.5kg/m^2^, yes vs no | 2.43 (1.47-4.00) | 2.95 (1.72-5.05) | 2.60 (1.56-4.33) |
|  | At least 1 assisted Activity of Daily Living, yes vs no | 2.87 (1.64-5.01) | 2.96 (1.64-5.32) | 2.65 (1.50-4.68) |
| **Prior healthcare utilization** | History of polyclinic visits in 6 months prior, yes vs no | - | - | 0.60 (0.37-0.97) |
|  | History of specialist outpatient visits in 6 months prior, yes vs no | 0.49 (0.31-0.77) | 0.44 (0.27-0.70) | 0.50 (0.31-0.79) |
| **Comorbidity** | History/diagnosis of cancer, yes vs no | 3.22 (1.60-6.46) | 3.50 (1.72-7.13) | 3.02 (1.48-6.18) |
|  | History/diagnosis of cerebrovascular disease, yes vs no | 2.40 (1.10-5.23) | 2.63 (1.19-5.80) | 2.34 (1.04-5.23) |
|  | History/diagnosis of diabetes (uncomplicated), yes vs no | - | - | 1.72 (0.95-3.12) |
|  | History/diagnosis of renal disease, yes vs no | - | - | 1.54 (0.82-2.90) |
|  |  |  |  |  |
|  | Area Under Curve | 0.74 (0.68, 0.79) | 0.77 (0.72, 0.82) | 0.74 (0.69, 0.79) |
|  | Intercept | -0.10 (-0.50, 0.30) | -0.13 (-0.51, 0.24) | -0.15 (-0.54, 0.23) |
|  | Slope | 0.93 (0.73, 1.14) | 0.91 (0.72, 1.10) | 0.90 (0.71, 1.10) |
|  |  |  |  |  |
|  | *At 0.10 threshold* |  |  |  |
|  | Sensitivity | 72% | 76% | 72% |
|  | Specificity | 65% | 68% | 67% |
|  | Positive predictive value | 22% | 25% | 24% |
|  | Negative predictive value | 94% | 95% | 95% |
|  |  |  |  |  |
|  | *At 0.30 threshold* |  |  |  |
|  | Sensitivity | 29% | 37% | 34% |
|  | Specificity | 95% | 95% | 95% |
|  | Positive predictive value | 46% | 48% | 49% |
|  | Negative predictive value | 91% | 92% | 91% |
|  |  |  |  |  |
|  | *At 0.50 threshold* |  |  |  |
|  | Sensitivity | 11% | 15% | 15% |
|  | Specificity | 99% | 99% | 99% |
|  | Positive predictive value | 60% | 66% | 61% |
|  | Negative predictive value | 89% | 89% | 89% |
|  |  |  |  |  |
|  | *At 0.70 threshold* |  |  |  |
|  | Sensitivity | 4% | 4% | 5% |
|  | Specificity | 100% | 100% | 100% |
|  | Positive predictive value | 71% | 59% | 84% |
|  | Negative predictive value | 88% | 88% | 88% |

LASSO: least absolute shrinkage and selection operator; OR: odds ratio
