# Supplementary figures and images for: The PROgnostic ModEl for chronic lung disease (PRO-MEL): development and temporal validation
Source: BMC Pulm Med. 2024 Aug 30;24:429. doi: 10.1186/s12890-024-03233-0 (PMC11365240; doi:10.1186/s12890-024-03233-0)

# Additional File 5. Calibration plots for final model for 3 imputed datasets (10th, 30th, 50th datasets)


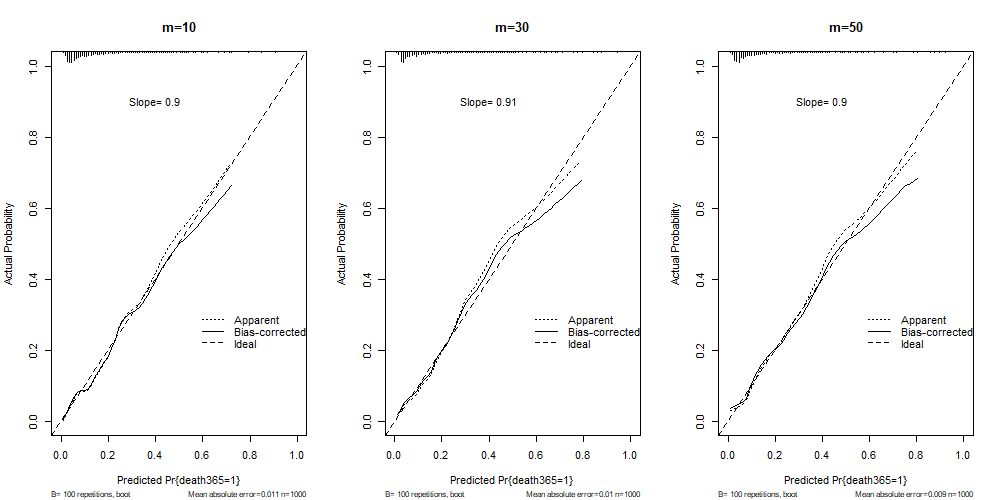

Supplement: Supplementary file 5 — Supplementary Material 5 [file 12890_2024_3233_MOESM5_ESM.docx]
