# Supplementary material for: The PROgnostic ModEl for chronic lung disease (PRO-MEL): development and temporal validation
Source: BMC Pulm Med. 2024 Aug 30;24:429. doi: 10.1186/s12890-024-03233-0 (PMC11365240; doi:10.1186/s12890-024-03233-0)
Supplement: Supplementary file 6 — Supplementary Material 6 [file 12890_2024_3233_MOESM6_ESM.docx]

# Additional File 6. Stability plots for final model for 3 imputed datasets (10^th^, 30^th^, 50^th^ datasets)

| m=10 | m=20 | m=30 |
| --- | --- | --- |


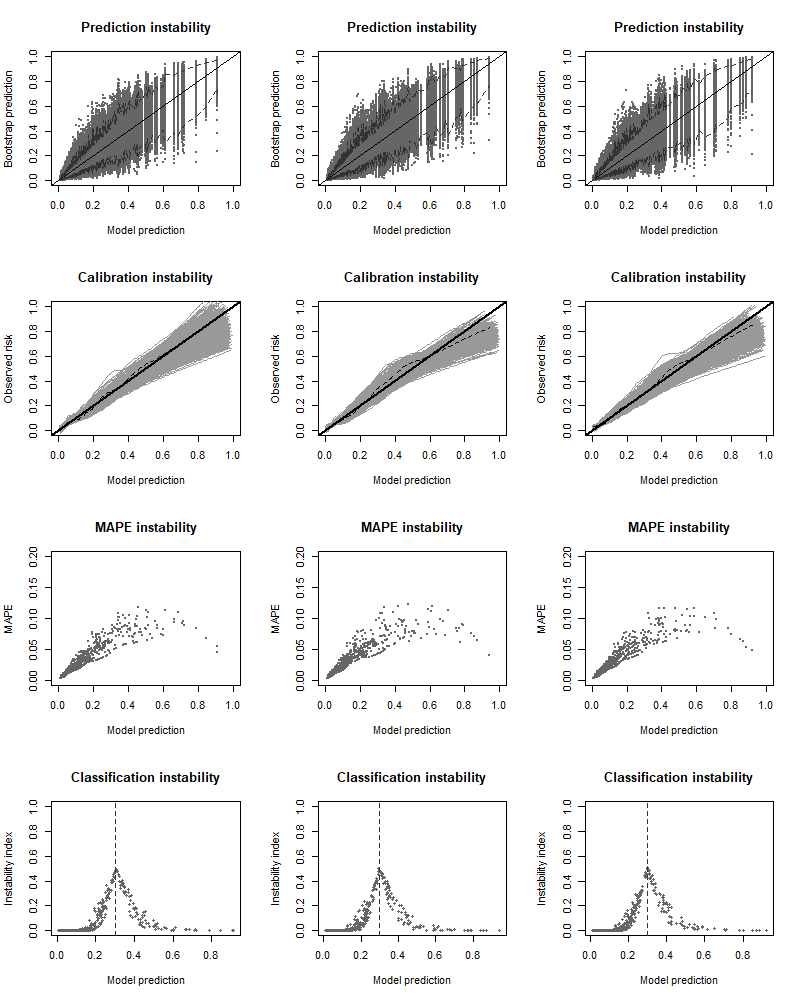


MAPE: Mean absolute percentage error. Plots illustrate the stability of model predictions, calibration curves, predicted errors and % misclassified (at a score cut-off of 0.3) respectively, over 1000 bootstrap samples of three imputed datasets.
