# Supplementary material for: The PROgnostic ModEl for chronic lung disease (PRO-MEL): development and temporal validation
Source: BMC Pulm Med. 2024 Aug 30;24:429. doi: 10.1186/s12890-024-03233-0 (PMC11365240; doi:10.1186/s12890-024-03233-0)
Supplement: Supplementary file 7 — Supplementary Material 7 [file 12890_2024_3233_MOESM7_ESM.docx]

# Additional File 7. PRO-MEL risk equation

The prognostic equation corresponding to the final model was:

| $Probability of death=1/(1+exp \left( -L \right), where$  $L= -5.51 + 0.03*Age +$  $0.57*\left( if male gender \right)+ 0.71*(if minority ethnic group)+$  $1.27*(if diagnosed with ILD) + 1.15* (if diagnosed with TB) +$  $1.56*\left( if started long term oxygen therapy \right)+$  $1.08*(if BMI<18.5) +1.08*(if at least 1 assisted ADL) +$  $1.25*(if history or diagnosis of cancer) +$  $0.97*(if history or diagnosis of cerebrovascular disease)$  $-0.82*\left( if history of SOC visits in 6 months prior \right),$  $where p is the probability of death within 1 year$ |
| --- |
