# Supplementary material for: The PROgnostic ModEl for chronic lung disease (PRO-MEL): development and temporal validation
Source: BMC Pulm Med. 2024 Aug 30;24:429. doi: 10.1186/s12890-024-03233-0 (PMC11365240; doi:10.1186/s12890-024-03233-0)
Supplement: Supplementary file 8 — Supplementary Material 8 [file 12890_2024_3233_MOESM8_ESM.docx]

# Additional File 8. Comparison of development and validation cohort characteristics

|  |  | **Development cohort** | **Validation cohort** | **p-value*** |
| --- | --- | --- | --- | --- |
| **Number of patients** |  | **1,000** | **500** |  |
| **Mortality at 1 year** |  | **12.2%** | **19.4%** |  |
|  |  |  |  |  |
| **Demographics (at index visit)** | | | | |
| Age, years | Median (Q1-Q3) | 72 (64-80) | 75 (68-82) | <0.001 |
|  |  |  |  |  |
| Gender | Female | 33.3 | 19.2 | <0.001 |
|  | Male | 66.7 | 80.8 |  |
|  |  |  |  |  |
| Marital status | Married | 33.9 | 39.6 | <0.001 |
|  | Single | 3.4 | 4.4 |  |
|  | Divorced/Widowed | 1.9 | 2.8 |  |
|  | Unknown | 60.8 | 53.2 |  |
|  |  |  |  |  |
| Racial minority | Minority | 18.9 | 19.8 | 0.68 |
|  |  |  |  |  |
| Housing type | 1-3 room flats | 35.0 | 41.0 | 0.06 |
|  | 4-5 room flats | 40.6 | 39.6 |  |
|  | Executive/ Multi-generation flats | 4.0 | 3.0 |  |
|  | Private and other housing | 20.4 | 16.4 |  |
|  |  |  |  |  |
| Smoking status | Ex-Smoker | 9.2 | 18.0 | <0.001 |
|  | Non-Smoker | 37.4 | 30.0 |  |
|  | Smoker | 26.9 | 38.4 |  |
|  | Unknown | 26.5 | 13.6 |  |
|  |  |  |  |  |
| **Lung disease characteristics (at index visit)** | | | | |
| Had a prior CLD diagnosis in past 5 years |  | 64.6 | 81.8 | <0.001 |
|  |  |  |  |  |
| Duration of disease (days) | Median (Q1-Q3) | 949.5 (373.5-1544) | 1,404 (629-1763) | <0.001 |
|  |  |  |  |  |
| Case setting of index visit | Inpatient | 6.3 | 24.6 | <0.001 |
|  | Emergency attendance | 33.9 | 27.8 |  |
|  | Day surgery | 0.3 | 0.2 |  |
|  | Specialist outpatient | 59.5 | 47.4 |  |
|  |  |  |  |  |
| LOS of index visit |  | 5 (3-15.5) | 7 (4-13) | 0.32 |
|  |  |  |  |  |
| Diagnosis of index visit |  |  |  |  |
| Bronchitis, emphysema or COPD |  | 55.0 | 80.6 | <0.001 |
| Bronchiectasis |  | 38.3 | 19.4 | <0.001 |
| Interstitial pulmonary diseases |  | 11.5 | 5.6 | <0.001 |
| >1 diagnosis at index visit |  | 5.9 | 5.6 | 0.91 |
|  |  |  |  |  |
| Respiratory complications |  |  |  |  |
| Chronic respiratory failure |  | 2.5 | 3.4 | 0.32 |
| Pulmonary hypertension or heart disease |  | 1.3 | 4.4 | <0.001 |
| Sequelae of respiratory and unspecified tuberculosis |  | 4.6 | 2.8 | 0.12 |
|  |  |  |  |  |
| **Comorbidity (Index visit + 1 year prior)** | | | | |
| Charlson Comorbidity Index score | Median (Q1-Q3) | 4 (3-7) | 5 (4-8) | <0.001 |
| Renal disease |  | 9.8 | 15.6 | 0.001 |
| Diabetes, uncomplicated |  | 16.6 | 16.2 | 0.88 |
| Diabetes, complicated |  | 10.7 | 16.6 | 0.002 |
| Cerebrovascular disease |  | 5.1 | 5.6 | 0.71 |
| Cardiac (CHF, MI, Arrhythmia) |  | 11.5 | 20.6 | <0.001 |
| Cancer |  | 5.5 | 7.4 | 0.17 |
| Anaemia |  | 11.0 | 15.8 | 0.01 |
| Dyslipidaemia |  | 31.9 | 35.2 | 0.20 |
| Hypertension |  | 39.3 | 45.6 | 0.02 |
|  |  |  |  |  |
| **Pulmonary history/parameters (Index visit + admissions within 6 months prior)** | | | | |
| Oxygen saturation <95% |  | 11.5 | 19.6 | 0.01 |
| Started long term oxygen therapy |  | 2.6 | 10.6 | <0.001 |
| Started invasive ventilation |  | 0.8 | 2.2 | 0.03 |
| Started acute invasive ventilation |  | 2.3 | 6.4 | <0.001 |
| Started long term non-invasive ventilation |  | 0.3 | 0.2 | 1.00 |
| MMRC Dyspnea Score ≥2 |  | 12.3 | 25.0 | 0.01 |
| **Biomarkers (Index visit + most recent admission in 6 months prior)** | | | | |
| Eosinophils, x10^9^/L | Median (Q1-Q3) | 0.1 (0-0.3) | 0.1 (0-0.3) | 0.74 |
| Neutrophils, x10^9^/L | Median (Q1-Q3) | 6.1 (4.4-8.7) | 6.7 (5.1-9.3) | <0.001 |
| MDRD, mL/min/1.73m^2^ | Median (Q1-Q3) | 73.3 (57.1-89.9) | 71 (53.5-87.2) | 0.13 |
| **Functional/physiological measurements (Index visit + most recent admission in 6 months prior)** | | | | |
| BMI, kg/m^2^ | Median (Q1-Q3) | 21.2 (18.5-24.6) | 21.3 (18.1-24.5) | 0.92 |
| BMI, Asian categories | <18.5 kg/m^2^ | 19.0 | 21.0 | <0.001 |
|  | 18.5-22.9 kg/m^2^ | 30.1 | 27.2 |  |
|  | 23.0-27.4 kg/m^2^ | 18.6 | 18.6 |  |
|  | ≥27.5 kg/m^2^ | 8.8 | 8.8 |  |
|  | NA | 23.5 | 24.4 |  |
| 1 or more assisted ADLs |  | 13.1 | 26.0 | <0.001 |
| 1 or more dependency in ADL |  | 1.9 | 2.2 | 0.85 |
| **Utilization (1 year prior)** | | | | |
| 1 or more inpatient (emergency) admissions |  | 36.8 | 59.2 | <0.001 |
| 1 or more respiratory (emergency) admission |  | 16.1 | 38.8 | <0.001 |
| 1 or more admission to HD/ICU |  | 6.1 | 14.2 | <0.001 |
| 1 or more inpatient (elective) admissions |  | 4.2 | 3.2 | 0.40 |
| 1 or more Emergency Department visits |  | 48.5 | 68.8 | <0.001 |
| 1 or more Specialist Outpatient Clinic visits |  | 81.3 | 86.8 | 0.01 |
| 1 or more day surgeries |  | 17.4 | 18.4 | 0.67 |
| 1 or more polyclinic visits |  | 48.1 | 46.2 | 0.51 |
| **End-of-life care** | | | | |
| Referral destination |  |  |  |  |
| Inpatient palliative department |  | 1.1 | 5.8 | <0.001 |
| Sub-acute care |  | 1.3 | 1.2 | 1.00 |

ADL: Activities of Daily Living; BMI: Body mass index; CHF: Congestive heart failure; CLD: chronic lung disease; COPD: Chronic obstructive pulmonary disease; HD/ICU: High dependency/ intensive care unit; MDRD GFR: Modification of Diet in Renal Disease glomerular filtration rate; MI: myocardial infarction; *Fisher's test and Wilcoxon ranksum tests were conducted when comparing categorical and continuous variables across groups respectively
